# Challenges for achieving safe and effective radical cure of *Plasmodium vivax*: a round table discussion of the APMEN Vivax Working Group

**DOI:** 10.1186/s12936-017-1784-1

**Published:** 2017-04-05

**Authors:** Kamala Thriemer, Benedikt Ley, Albino Bobogare, Lek Dysoley, Mohammad Shafiul Alam, Ayodhia P. Pasaribu, Jetsumon Sattabongkot, Elodie Jambert, Gonzalo J. Domingo, Robert Commons, Sarah Auburn, Jutta Marfurt, Angela Devine, Mohammad M. Aktaruzzaman, Nayeem Sohel, Rinzin Namgay, Tobgyel Drukpa, Surender Nath Sharma, Elvieda Sarawati, Iriani Samad, Minerva Theodora, Simone Nambanya, Sonesay Ounekham, Rose Nanti Binti Mudin, Garib Da Thakur, Leo Sora Makita, Raffy Deray, Sang-Eun Lee, Leonard Boaz, Manjula N. Danansuriya, Santha D. Mudiyanselage, Nipon Chinanonwait, Suravadee Kitchakarn, Johnny Nausien, Esau Naket, Thang Ngo Duc, Ha Do Manh, Young S. Hong, Qin Cheng, Jack S. Richards, Rita Kusriastuti, Ari Satyagraha, Rintis Noviyanti, Xavier C. Ding, Wasif Ali Khan, Ching Swe Phru, Zhu Guoding, Gao Qi, Akira Kaneko, Olivo Miotto, Wang Nguitragool, Wanlapa Roobsoong, Katherine Battle, Rosalind E. Howes, Arantxa Roca-Feltrer, Stephan Duparc, Ipsita Pal Bhowmick, Enny Kenangalem, Jo-Anne Bibit, Alyssa Barry, David Sintasath, Rabindra Abeyasinghe, Carol H. Sibley, James McCarthy, Lorenz von Seidlein, J. Kevin Baird, Ric N. Price

**Affiliations:** 1 Global and Tropical Health Division, Menzies School of Health Research and Charles Darwin University, Darwin, Australia; 2 National Vector Borne Disease Control Programme, Honiara, Solomon Islands; 3 National Center for Parasitology, Entomology and Malaria Control, Phnom Penh, Cambodia; 4 School of Public Health, National Institute of Public Health, Phnom Penh, Cambodia; 5 International Centre for Diarrheal Diseases and Research, Dhaka, Bangladesh; 6 Department of Pediatrics, Medical Faculty, University of Sumatera Utara, Medan, Indonesia; 7 Mahidol Vivax Research Unit, Faculty of Tropical Medicine, Mahidol University, Bangok, Thailand; 8 Medicines for Malaria Venture (MMV), Geneva, Switzerland; 9 Diagnostics Program, PATH, Seattle, USA; 10 Mahidol Oxford Tropical Medicine Research Unit (MORU), Bangok, Thailand; 11 Centre for Tropical Medicine and Global Health, Nuffield Department of Medicine, University of Oxford, Oxford, UK; 12 Ministry of Health and Family Welfare, Malaria and Parasitic Disease Control, Director General of Health Services, Mohakhali, Dhaka, Bangladesh; 13 Vector-borne Disease Control Programme (VDCP), Department of Public Health, Ministry of Health in Bhutan, Thimphu, Bhutan; 14 National Vector Borne Disease Control Programme Directorate General of Health Services Ministry of Health & Family Welfare, New Delhi, India; 15 National Malaria Control Program, Jakarta, Indonesia; 16 National Center for Entomology Parasitology and Malaria Control, Vientiane, Laos; 17 Vector-borne Diseases Control Programme, Ministry of Health Malaysia, Kuala Lumpur, Malaysia; 18 Malaria Control Programme, Epidemiology and Disease Control Division, Department of Health Services, Ministry of Health and Population, Kathmandu, Nepal; 19 National Malaria Control Programme, Goroka, Papua New Guinea; 20 Malaria Control Programme, Department of Health, Manila, Philippines; 21 Division of Malaria & Parasitic Diseases, Korea Centers for Disease Control and Prevention, Seoul, South Korea; 22 Antimalaria Campaign, Ministry of Health, Colombo, Sri Lanka; 23 Bureau of Vector-Borne Disease, Department of Disease Control, Ministry of Public Health, Bangkok, Thailand; 24 National Malaria Control Programme, Port Vila, Vanuatu; 25 National Institute of Malaria, Parasitology, and Entomology (NIMPE), Tam Kỳ, Vietnam; 26 Access Bio Inc, Monmouth, USA; 27 Australian Army Malaria Institute, Enoggera, QLD Australia; 28 Centre for Biomedical Research, Burnet Institute, Melbourne, Australia; 29 Vector Borne Disease Control, Ministry of Health, Jakarta, Indonesia; 30 Indonesian Parasitic Association, Jakarta, Indonesia; 31 Eijkman Institute of Molecular Biology, Jakarta, Indonesia; 32 FIND, Geneva, Switzerland; 33 Jiangsu Institute of Parasitic Diseases, Wuxi, China; 34 Karolinska Institutet, Stockholm, Sweden; 35 Osaka City University, Osaka, Japan; 36 Centre for Genomics and Global Health, Wellcome Trust Centre for Human Genetics, University of Oxford, Oxford, UK; 37 Wellcome Trust Sanger Institute, Hinxton, UK; 38 Department of Molecular Tropical Medicine, Faculty of Tropical Medicine, Mahidol University, Salaya, Thailand; 39 Malaria Atlas Project, Nuffield Department of Medicine, Oxford Big Data Institute, University of Oxford, Oxford, UK; 40 Malaria Consortium, London, UK; 41 Model Rural Health Research Unit (MRHRU), Tripura State, ICMR, Agartala, India; 42 Yayasan Pengembangan Kesehatan dan Masyarakat Papua Timika, Timika, Indonesia; 43 The Research Institute for Tropical Medicine, Manila, Philippines; 44 The Walter + Eliza Hall Institute of Medical Research, Melbourne, Australia; 45 Department of Medical Biology, University of Melbourne, Parkville, VIC Australia; 46 President’s Malaria Initiative, United States Agency for International Development, Bangkok, Thailand; 47 World Health Organization Western Pacific Region, Manila, Philippines; 48 WWARN, Oxford, UK; 49 University of Washington, Seattle, WA USA; 50 QIMR Berghofer Medical Research Institute, Brisbane, Australia; 51 Eijkman-Oxford Clinical Research Unit, Jakarta, Indonesia

**Keywords:** Vivax malaria, *P. vivax*, Radical cure, Primaquine, APMEN, Tafenoquine

## Abstract

The delivery of safe and effective radical cure for *Plasmodium vivax* is one of the greatest challenges for achieving malaria elimination from the Asia–Pacific by 2030. During the annual meeting of the Asia Pacific Malaria Elimination Network Vivax Working Group in October 2016, a round table discussion was held to discuss the programmatic issues hindering the widespread use of primaquine (PQ) radical cure. Participants included 73 representatives from 16 partner countries and 33 institutional partners and other research institutes. In this meeting report, the key discussion points are presented and grouped into five themes: (i) current barriers for glucose-6-phosphate deficiency (G6PD) testing prior to PQ radical cure, (ii) necessary properties of G6PD tests for wide scale deployment, (iii) the promotion of G6PD testing, (iv) improving adherence to PQ regimens and (v) the challenges for future tafenoquine (TQ) roll out. Robust point of care (PoC) G6PD tests are needed, which are suitable and cost-effective for clinical settings with limited infrastructure. An affordable and competitive test price is needed, accompanied by sustainable funding for the product with appropriate training of healthcare staff, and robust quality control and assurance processes. In the absence of quantitative PoC G6PD tests, G6PD status can be gauged with qualitative diagnostics, however none of the available tests is currently sensitive enough to guide TQ treatment. TQ introduction will require overcoming additional challenges including the management of severely and intermediately G6PD deficient individuals. Robust strategies are needed to ensure that effective treatment practices can be deployed widely, and these should ensure that the caveats are outweighed by  the benefits of radical cure for both the patients and the community. Widespread access to quality controlled G6PD testing will be critical.

## Background

In November 2014, the governments of the Asia–Pacific nations reconfirmed their commitment to the regional elimination of malaria by 2030. Although major gains in malaria control have been made over the last two decades, these successes have been far less apparent for *Plasmodium vivax* than for *Plasmodium falciparum*. Once regarded as a relatively benign disease, vivax malaria is now acknowledged as an important public health concern leading to life-threatening complications, miscarriage, chronic infection and increased mortality [1–3]. *P. vivax* poses specific difficulties to elimination, mainly due to its ability to relapse weeks to months after the initial infection [4]. The propensity of *P. vivax* to form dormant liver stages (hypnozoites) leading to recurrent infections, requires specific strategies to achieve its elimination, including the provision of radical cure to treat both the blood and dormant liver stages of the parasite.

The only widely available drug to treat hypnozoites is primaquine (PQ), a drug which can cause haemolysis when administered to patients with a glucose-6-phosphate-dehydrogenase (G6PD) enzyme deficiency [5]. G6PD deficiency (G6PDd) is the most common enzymopathy worldwide, with more than 185 clinically relevant G6PD variants reported [6], conferring varying degrees of phenotypic deficiency. The gene encoding the G6PD enzyme is located on the X-chromosome, hence males can be hemizygous normal (wildtype) or deficient, whereas women can be homozygous-, heterozygous-deficient or normal for the G6PD variants. Heterozygous females harbour two distinct populations of red blood cells (RBCs), a G6PD normal and a G6PDd fraction. In heterozygous females the distribution of G6PDd and G6PD normal RBCs is determined at random through a process called lyonization [7]. Accordingly, heterozygous females with the same G6PD alleles can manifest different degrees of deficiency.

As a result of these factors, the risk of haemolysis varies with the dose of PQ administered, the level of G6PD enzyme activity and the genetic variant of G6PDd. The World Health Organization’s (WHO) malaria treatment guidelines recommend that PQ is administered over 14 days to reduce the risk of severe haemolysis [8], but such a prolonged treatment course poses significant issues regarding adherence that limits the regimens’ effectiveness. Tafenoquine (TQ), another 8-aminoquinoline compound which is currently at the end of it's Phase 3 clinical programme has a significantly longer half-life than PQ, allowing it to be administered as a single dose regimen. Like PQ, TQ can cause severe haemolysis in G6PDd individuals, and it will therefore be crucial to determine the G6PD status prior to prescribing the drug to mitigate the risk of sustained haemolysis (Justin Green, pers. comm.).

Currently only 7 malaria-endemic countries in the Asia–Pacific region and Sri Lanka, which is malaria free, recommend G6PD testing prior to PQ treatment (Table 1) [9]. However few countries have introduced G6PD testing into routine practice due to considerable barriers for its public health deployment [10].

**Table 1:** Countries in the APMEN region and their recommendation in regards to vivax treatment and G6PD testing prior to PQ administration, based on WHO’s World Malaria Report, 2016 [9]

Country	Current treatment recommendation for *P. vivax*	Year PQ adopted	G6PD testing recommended in guidelines	Year policy on G6PD testing adopted
Bangladesh	CQ + PQ (14 days at 0.25 mg/kg)	2008	No	
Bhutan	CQ + PQ (14 days at 0.25 mg/kg)	Unknown	No	
Cambodia	DHA-PPQ + PQ (14 days at 0.25 mg/kg)	2013	Yes	2012
China	CQ + PQ (8 days at 0.75 mg/kg)	1970	No	
Democratic People’s Republic of Korea	CQ + PQ (14 days at 0.25 mg/kg)	2000	No	
India	CQ + PQ (14 days at 0.25 mg/kg)	1982	No	
Indonesia	DHA-PPQ + PQ (14 days at 0.25 mg/kg)	2004	No	
Lao People’s Democratic Republic	CQ + PQ (14 days at 0.25 mg/kg)	Unknown^a^	Yes	2010
Malaysia	CQ + PQ (14 days at 0.5 mg/kg)	1993	Yes	1993
Myanmar	CQ + PQ (14 days at 0.25 mg/kg)	1951	No	
Nepal	CQ + PQ (14 days at 0.25 mg/kg)	2009	Yes	Unknown
Papua New Guinea	AL + PQ (14 days at 0.25 mg/kg)	2009	No	
Philippines	CQ + PQ (14 days)	2002 or 2007^b^	Yes	2009
Republic of Korea	CQ + PQ (14 days at 0.25 mg/kg)	2001	No	
Solomon Islands	AL + PQ (14 days at 0.25 mg/kg)	2009	Yes	2009
Sri Lanka^c^	CQ + PQ (14 days at 0.25 mg/kg)	Unknown	Yes	Unknown
Thailand	CQ + PQ (14 days at 0.25 mg/kg)	1965	Yes	2015
Timor-Leste	CQ + PQ (14 days at 0.5 mg/kg)	2006	No	
Vanuatu	AL + PQ (14 days at 0.25 mg/kg)	2009	Yes	2009
Vietnam	CQ + PQ (14 days at 0.25 mg/kg)	1960	No	

^a^WHO report states “no” in the respective section, but PQ included in current guidelines

^b^Unclear from WHO report

^c^data from WHO world malaria report 2015 [57], since Sri Lanka is not anymore included in the 2016 report

## The Asia Pacific Malaria Elimination Network and the Vivax Working Group

The Asia Pacific Malaria Elimination Network (APMEN) in conjunction with the Asia Pacific Malaria Alliance (APLMA) is a regional network of National Malaria Control Programmes (NMCPs) and research partners working together to overcome the challenges for the regional elimination of malaria. At the initial APMEN meeting in 2009, *P. vivax* was identified as a key challenge for the regional malaria elimination. The Vivax Working Group (VxWG) was established to identify key knowledge gaps impeding the control of vivax malaria [11]. The VxWG provides a forum for its members to prioritise research activities that will provide the necessary evidence for policy makers to change policy and impact on health outcomes. The group comprises representatives from 18 NMCPs, a wide range of research partner institutes, the WHO, as well as a variety of consortia and industry representatives. The working group follows a cyclical process as described in detail previously [11]. Annual workshops, meetings and consultations are a critical part of the groups work to build consensus, set common agendas and foster partnerships.

## Round table discussion “Incorporation of G6PD testing for *P. vivax* case management, how and when to use it”

The annual VxWG meeting was held in October 2016 in Bali Indonesia with 73 representatives from 16 partner countries and 33 institutional partners and other research institutes. During this meeting a round table discussion was held to discuss the incorporation of G6PD testing for *P. vivax* case management and how and when to use it. The specific questions posed to the forum are listed in Table 2. The key discussion points arising from this session are grouped into five overarching issues: (i) current barriers for G6PD testing for PQ radical cure, (ii) necessary properties of G6PD tests for wide scale deployment, (iii) the promotion of G6PD testing, (iv) improving the adherence to a complete PQ treatment course and (v) challenges for future TQ roll out.

**Table 2:** Questions posed to participants for the round table discussions

1	Should G6PD testing *always* be done prior to prescribing primaquine? (What does the current WHO recommendation mean for your program?)
2	What are the key barriers for introducing routine G6PD testing? (e.g. barriers at decision maker level, at provider level)
3	How can we promote G6PD testing prior to primaquine or tafenoquine? (e.g. what evidence is needed to make a case for testing, how can it be funded, what should it cost, what support is needed?)
4	What will be the challenges rolling out tafenoquine?
5	How would you provide G6PD testing when tafenoquine is rolled out? (E.g. at what level? Who will test? How will the results be recorded? Testing before every episode? Are there areas with high P.v. burden where you believe G6PD testing would not be feasible? If so, what alternatives could be considered?)
6	How can we encourage primaquine usage for radical cure?
7	How can we improve treatment adherence? (e.g. are there specific issues with adherence in hard to reach populations and how to solve them?)
8	What kinds of tests do we need for routine G6PD testing? (e.g. test format, operational characteristics, training involved, cost per test etc.)

## Topic 1: current barriers for G6PD testing for PQ radical cure

The WHO malarial treatment guidelines were revised recently, including a statement that good practice requires that the G6PD status of patients should be ascertained prior to administration of PQ [8]. Implementing routine testing for G6PDd is challenging in the absence of a robust, affordable point of care (PoC) test and is not yet universally accepted. Hence the WHO guidelines also state that if testing is unavailable, an individual risk–benefit assessment should guide the decision on whether or not to prescribe PQ (Table 3) [8].

**Table 3:** Relevant section from WHO guidelines on G6PD testing for PQ based radical cure [8]

Statement	Section
The G6PD status of patients should be used to guide administration of PQ for preventing relapse. Good practise statement	Executive summary—page 11 Treatment of uncomplicated malaria caused by *P. vivax, P. ovale, P. malariae or P. knowlesi*—page 60
When G6PD status is unknown and G6PD testing is not available, a decision to prescribe PQ must be based on an assessment of the risks and benefits of adding PQ. Good practise statement	Executive summary—page 11 Treatment of uncomplicated malaria caused by *P. vivax, P. ovale, P. malariae or P. knowlesi*—page 60
Given the benefits of preventing relapse and in the light of changing epidemiology worldwide and more aggressive targets for malaria control and elimination, the group now recommends that PQ be used in all settings	Treatment of uncomplicated malaria caused by *P. vivax, P. ovale, P. malariae or P. knowlesi*—page 68
In the absence of quantitative testing, all females should be considered as potentially having intermediate G6PD activity and given the 14-day regimen of PQ, with counselling on how to recognize symptoms and signs of haemolytic anaemia	Treatment of uncomplicated malaria caused by *P. vivax, P. ovale, P. malariae or P. knowlesi*—page 69
If G6PD testing is not available, a decision to prescribe or withhold PQ should be based on the balance of the probability and benefits of preventing relapse against the risks of PQ induced haemolytic anaemia. This depends on the population prevalence of G6PD deficiency, the severity of the prevalent genotypes and on the capacity of health services to identify and manage PQ induced haemolytic reactions	Treatment of uncomplicated malaria caused by *P. vivax, P. ovale, P. malariae or P. knowlesi*—page 69

National policies vary considerably from country to country, with only 7 of the 20 malaria-endemic countries in the Asia–Pacific region and Sri Lanka, which is malaria free, currently recommending testing for G6PDd prior to prescribing PQ (Table 1) [9]. In some countries it was acknowledged that the actual clinical practise differs from the guidelines. Identifying and addressing the barriers for the introduction of G6PD testing will help to promote the safe delivery of PQ. Furthermore, since G6PD testing will likely be a prerequisite for TQ prescription, which may demand far more stringent criteria, ensuring the widespread implementation of G6PD testing will greatly facilitate the roll out of this new treatment once it becomes available.

Many participants questioned the need for routine G6PD testing prior to PQ radical cure, perceiving the risk of drug induced severe haemolysis to be low, and citing extensive experience in treating patients with PQ with very few reports of severe adverse events. This perception was echoed in a more formal study undertaken in four of the APMEN partner countries [10]. Many participants proposed that in certain populations and locations it would be safe to prescribe PQ without prior G6PD testing.

A counter argument to this approach was raised that, since there was a lack of formal pharmacovigilance systems the occurrence of severe adverse events would be rarely recognised or recorded. A comprehensive review of reported PQ induced toxicity documented an overall risk of mortality as 1 in 621,428 [12]. However there are several reports of severe PQ toxicity and in some cases these were fatal [13, 14]. Most of these reports were not accompanied by corresponding denominators for the number of patients exposed and hence there is a degree of uncertainty about the absolute risk of haemolysis and how this varies with different G6PD variants. The Mediterranean and Mediterranean-like G6PDd variants are of particular concern because of severe PQ induced haemolytic anaemia that is not self-limiting [15].

The perceived need for G6PD testing therefore depends on a balance of the population-level risk of drug induced haemolysis and the benefits of achieving radical cure. The healthcare priority for managing a patient with acute malaria is the reduction in asexual parasitaemia and achieving symptom resolution. In this context the prescriber and/or patient may not perceive the immediate expediency of preventing future relapse which occur in only a proportion of patients and often do not manifest clinically for weeks or even months [16]. Each recurrent bout of parasitaemia is associated with parasite induced haemolysis and haemopoietic suppression resulting in a cumulative risk of severe anaemia [17]. The morbidity and mortality associated with recurrent infections will vary between geographical populations, access to healthcare, and the prevailing relapse patterns [18]. Hence the risk–benefit assessment varies, depending on the clinical setting and the specific application of PQ, such as the treatment of symptomatic patients, terminal prophylaxis in healthy but exposed individuals and even mass drug administration [19, 20]. The risk–benefit also depends on the progress of malaria elimination in the respective setting. In countries with low endemicity close to malaria elimination the risk of reinfection is low and reappearance of parasites is mainly caused by relapses rendering radical cure highly beneficial. The ability of the health system to ensure adherence to treatment is an additional variable that should be included in the risk–benefit assessment, since incomplete treatment only provides the risks, but not the benefits of radical cure.

The logistics of delivering diagnostics in many malaria endemic areas are substantial. At the round table discussions participants raised concerns regarding the cost-effectiveness of G6PD testing in areas with low risk of PQ induced haemolysis. In countries with limited public health resources this issue becomes crucial for implementation routine G6PD testing and policy makers must be convinced that G6PD testing adds value and is good use of available funding. Economic studies to address this are currently underway in a number of Asian countries [21]. Preliminary modelling data from the Thai-Myanmar border suggests that there is a potential reduction in total healthcare costs when using G6PD testing prior to PQ compared to PQ treatment without testing. These savings result from averting drug induced haemolytic episodes and the cumulative impact of *P. vivax* relapses [22].

Participants from NMCPs highlighted the financial challenges, and the barriers of ensuring appropriate training and quality control practices. Furthermore the logistical challenges of maintaining a supply chain and suitable storage facilities are paramount in order to use tests within their expiry dates. This is especially important for low endemic settings where only a few vivax patients are seen, but stocks would need to be kept up to date.

Wide-scale introduction of G6PD testing for vivax patients will require the availability of easy to use, reliable PoC tests with robust performance indicators [6]. Several participants discussed the difficulties of introducing malaria Rapid Diagnostic Tests (RDTs) including the practice of adhering to guidelines when the RDT result was negative [23–25] and the variable utilization in some settings [26–30]. Although the lessons learnt from the introduction of malaria RDTs are relevant to G6PD testing, the former are required for diagnosing an acute infection, whereas the latter are required for safety and prevention of future infections [31], and thus the experience and challenges are often different.

## Topic 2: necessary properties of G6PD test for wide scale deployment

Most endemic countries where G6PD testing is carried out routinely rely on the fluorescent spot test (FST) as the primary diagnostic tool. In some cases the FST is backed up by spectrophotometer based quality control systems (e.g. Malaysia). Whilst the FST is widely used, it has a number of limitations including difficulties in its interpretation, the need for basic laboratory infrastructure and an extended time to result of at least 30–45 min, rendering the test unsuitable for PoC testing [6]. The FST has a cut-off enzyme activity at approximately 30% of the adjusted male median [32]. Whilst this threshold is currently widely believed to be suitable to guide PQ treatment, it is not sufficient to guide TQ treatment and the manufacturers recommendations will likely require an individual’s enzyme activity to be greater than 70% [5, 33].

The FST provides a qualitative test result and is unable to diagnose heterozygous females who can have G6PD activity between 30 and 70%. In heterozygous females the fraction of RBCs with G6PD normal activity produces a G6PD normal result, however following PQ administration, the fraction of G6PDd RBCs will be subject to drug induced haemolysis, which can result in a substantial drop in haemoglobin. In settings where the FST is already used as a routine diagnostic, there is no immediate need for a change of practice to other tests as long as medical staff is aware of the test’s shortcomings in heterozygous females.

Two test formats have been introduced to the market within recent years. One of these is a lateral flow assay based on a colorimetric reaction that provides a qualitative G6PD result within less than 15 min. The most widely used lateral flow assay is the CareStart G6PD RDT (Accessbio, USA) with operational characteristics suitable for application in the field [6]. The test has been evaluated thoroughly and in most cases found to perform comparably to the FST [34–38]. It has a similar cut-off activity to the FST and hence it is not suitable for guiding treatment with TQ [6] however its superior operational characteristics and price [6, 37] make it a useful alternative to the FST prior to PQ treatment.

The second test format is a quantitative biosensor (Accessbio, USA). The Biosensor measures electrochemical properties of a blood sample and provides a quantitative G6PD activity reading that requires normalizing by a haemoglobin measurement or RBC count. While the performance of the current biosensor is not yet sufficient to replace the FST, the format addresses two important shortcomings of all other currently available PoC tests [39]. The machine can provide a quantitative reading making it adaptable to different test-and-treat scenarios in which drug therapy may be based on different enzyme cut-offs. Unlike the lateral flow format the biosensor can identify heterozygous females with G6PD activities between 30 and 70% of the adjusted male median [32]. Some participants had concerns that the quantitative outcome was too complex for basic field applications. However it may be possible to convert the biosensor outcome into a qualitative reading according to predefined absolute cut-off activities.

Next generation biosensors are being developed which will have improved performance and if successful these will facilitate greatly the transition from PQ to TQ based radical cure. This advance in diagnostics is likely to come at greater financial costs since the biosensor and corresponding supplies are significantly more expensive (500 USD/machine, 2.50 USD/test) than the CareStart G6PD RDT (1.50 USD/test) and the FST (<$1/test) [6].

## Topic 3: promoting the roll out of G6PD testing

Participants discussed the criteria that need to be met before NMCPs can endorse G6PD testing to support PQ based radical cure. These were: (i) a clear risk–benefit and cost-effectiveness assessment of G6PD testing, (ii) the availability of a robust tests that is reliable in field settings with limited infrastructure, (iii) a competitive and affordable price for the test and (iv) secured and sustainable funding for the test, for training of health care staff and associated quality control processes. The first three points were discussed in topics 1 and 2 above.

Appropriate training of health care workers and laboratory personnel is needed at all relevant levels of the health system, with an emphasis on the management of G6PDd patients and appropriate monitoring on adherence to guidelines. Successful introduction and scale up of testing will require a system of quality assurance and monitoring mechanisms that should start at the moment of sample collection. This would also need to encompass the correct recording and reporting of results and subsequent adherence to respective treatment algorithms.

Country partners mentioned significant logistical challenges regarding the introduction of routine G6PD testing and emphasised the lack of evidence on how routine G6PD testing could be achieved nationally. Several participants called for pilot projects where efforts could be made to identify and address logistical bottlenecks and provide reassurance of feasibility. In a pilot project in Thailand CareStart™ G6PD RDTs were deployed in 62 selected malaria clinics in 16 provinces (personal communication Suravadee Kitchakam). The main challenges encountered were around the procurement process, the training of staff, the interpretation of test results and following treatment algorithms. Lessons learned from this kind of pilot studies will be help NMCPs to focus resources to achieve widespread deployment.

## Topic 4: improving the adherence to a full course of primaquine treatment

Adherence to the currently recommended 14 day PQ regimen is challenging. Previous studies have shown that adherence to a full course of 14 days is often low [40–43] and, whilst this may be mitigated by a shorter treatment course [21], even unsupervised 7 days regimens may be compromised by poor adherence [44]. On the other hand, the experience with tuberculosis (TB) and HIV suggests that extended treatment courses are feasible. Directly observed treatment (DOT) is used to improve adherence to TB treatment and has been shown to improve outcomes for malaria as well [41, 42]. Many participants commented on the importance of context specific solutions, rather than a “one size fits all” approach. For example, in a very low endemic setting such as Malaysia with a relatively well sourced health system, it is feasible to admit all patients with malaria for treatment and this ensures close supervision and maximal treatment efficacy. In countries with greater case numbers, less well funded health systems or impoverished populations that rely on a daily income admitting all malaria patients to hospital is not feasible. DOTs programmes delivered by village workers might be an option in some settings as was recently discussed at a meeting in Cambodia [45], however this approach is not formally endorsed by WHO. Some participants suggested that incentives for village health workers would encourage them to remind patients to adhere to their treatment.

Adequate patient communication and simple messaging have been shown to overcome poor adherence in settings with low education and literacy rates [46]. Where *P. vivax* and *P. falciparum* are co-endemic some participants thought that it was important that patients were aware of the biomedical difference between the two species. Explaining the long-term risk of *P. vivax* repeated infections due to its propensity to recur and the beneficial effects of radical cure was considered essential in encouraging patients and their carers to complete a full course of treatment.

Alternative solutions were discussed including modern communication technologies such as messaging through mobile phones (SMS) or using specifically developed apps. Results from trials using SMS to increase adherence to malaria treatment and to treatment for other diseases such as HIV and TB have shown mixed results [47–50]. Content and type of messaging need to be well developed, tested and refined and locally adapted for their implementation to be successful [51, 52]. Mobile applications have been developed to improve adherence to a range of other diseases however there is little knowledge on whether they actually impact on adherence [53].

## Topic 5: anticipated challenges for rolling out Tafenoquine

TQ is currently at the end of its phase 3 development programme. Phase 3 read out anticipated in 2017 and subsequent licensing expected to follow in endemic countries as approvals are gained. If marketed, TQ as a single dose radical cure will be a major advance in improving the adherence issues associated with PQ regimens. However, TQs long elimination half-life means that if drug induced signs of haemolysis occur treatment can’t be curtailed by ceasing further drug administration as is currently the case with PQ. This is likely to be particularly important for patients with the Mediterranean variant in whom haemolysis continues without compensatory effect, but less important for those with mild or moderate variants.

Patients with G6PDd, particularly those with Mediterranean variants, may be at risk of an extended period of haemolysis and for this reason prior testing for G6PDd is likely to be mandatory. Until more information has been gathered from heterozygous females with intermediate G6PDd (enzyme activity between 30 and 70%) the licence holder will restrict its use to those with a minimum G6PD enzyme activity of 70%. Identification of patients at risk through a reliable quantitative PoC test will therefore be one of the greatest challenges for access to TQ [54]. Alternative solutions need to be considered in case no appropriate PoC test is available in time. Participants suggested that patients could be referred to centres where quantitative testing, for instance using spectrophotometry, could be assured. It seems likely that the licence holder will insist on such safety precautions to ensure an acceptable risk–benefit ratio. However restricting the availability of TQ to tertiary centres with quantitative G6PD measurement facilities will significantly limit the number of patients receiving TQ treatment, and thus the public benefits.

Other challenges that need to be addressed prior to the introduction of TQ were also discussed. Current testing and treatment of malaria is generally undertaken by healthcare and laboratory workers with limited training. Given the risk of sustained haemolysis following TQ, medical and laboratory personnel will require enhanced training on testing, interpretation of the results and treatment protocols, as well as ensuring appropriate quality control processes are in place. Alternatively, higher levels of care may need to be provided, including more physician involvement, however this is unlikely to be feasible for many NMCPs.

Once appropriate PoC G6PD tests are available, these will need to be introduced at all outlets where administration of TQ is envisaged. One option to facilitate this is to assess every vivax patient routinely prior to TQ treatment. Another option is to test patients only once, assuming no major changes in enzyme activity occur over a life-time, and to record the patient’s results on a register or patient card, which could be used to base decisions on treatment in the future. Whether records should be kept as hard or soft copy and whether results should remain with the patient or in a centralized repository will depend largely on the capacity of the NMCPs and the health care system in general. In Malaysia and the Philippines new-borns have their G6PD activity measured. The carers of the babies are provided with cards indicating the results. Those tested normal will need to be tested again later in life to confirm their status, as G6PD levels are physiologically elevated in new-borns [55, 56]. The potential loss of cards over time, may limit the usefulness of the system for many NMCPs [10]. In the absence of electronic patient record systems and ongoing uncertainty regarding the stability of enzyme activity over time in heterozygous females, it is likely that most control programs will opt for repeated testing.

Adherence to test results and clear decision trees for adequate treatment of G6PDd patients will need to be developed and staff will require training and regular assessment on how well they are adhering to these protocols. Training of staff will need to include quality assurance issues, appropriate handling of samples for testing, as well as adequate communication to patients about the prescription of TQ, its benefits and risks and alternate treatment options when TQ is contraindicated.

## Conclusion

There are significant challenges for achieving safe and effective radical cure in the communities at greatest risk of malaria. NMCPs, researchers and funders need to address these challenges and create a viable strategy to achieve their goals, providing novel solutions for overcoming critical bottle-necks. This process needs to begin now to enhance treatment practice for PQ based radical cure. Highlighting the benefits of radical cure for the patient and community will improve prescription practice and patient adherence. Coupling this with improved access to adequate G6PD testing will pave the way for the introduction of TQ, with huge potential to accelerate the elimination of *P. vivax*.
